# Interpretable decision trees through MaxSAT

**DOI:** 10.1007/s10462-022-10377-0

**Published:** 2022-12-27

**Authors:** Josep Alòs, Carlos Ansótegui, Eduard Torres

**Affiliations:** Logic & Optimization Group (LOG), University of Lleida, Lleida, Spain

**Keywords:** Decision trees, MaxSAT, Explainable AI

## Abstract

We present an approach to improve the accuracy-interpretability trade-off of Machine Learning (ML) Decision Trees (DTs). In particular, we apply Maximum Satisfiability technology to compute Minimum Pure DTs (MPDTs). We improve the runtime of previous approaches and, show that these MPDTs can outperform the accuracy of DTs generated with the ML framework *sklearn*.

## Introduction

Recently, there has been a growing interest in creating synergies between Combinatorial Optimization (CO) and Machine Learning (ML), and vice-versa. This is a natural connection since ML algorithms can be seen in essence as optimization algorithms that try to minimize prediction error. In this paper, we focus on how CO techniques can be applied to improve the interpretability and accuracy of decision tree classifiers in ML.

A Decision Tree (DT) classifier is a supervised ML technique that builds a tree-structured classifier, where internal nodes represent the features of a dataset, branches represent the decision rules and each leaf node represents the outcome. In essence, every path from the root to a leaf is a classification rule that determines to which class belongs the input query.

ML research has been traditionally concerned with developing accurate classifiers, i.e., classifiers whose prediction error is as low as possible. However, lately, the research community is looking at the trade-off between accuracy and interpretability, where the latter refers to the human ability to understand how the classifier works. Interpretability is fundamental in many senses: to explain ML models and understand their value and accuracy, to debug machine learning algorithms, to make more informed decisions, etc.

Among supervised learning techniques, decision tree classifiers are interpretable in itself, as long as they are *small*, i.e., as few rules as possible, and as short as possible. As stated in Bessiere et al. (2009), informally, this follows from the principle of Occam’s Razor that states that one should prefer simpler hypotheses. However, notice that this is just one of the diverse aspects that may have to do with interpretability at least from a human perspective.

CO approaches have been applied to compute *small* trees in the sense of building decision trees of minimum size (number of nodes) or minimum depth. Minimizing the size corresponds indirectly to minimizing the number of rules associated with the DT and minimizing the depth corresponds to minimizing the length of the rules. Among these approaches we find several contributions coming from Operation Research (OR) (Aglin et al. 2020; Verwer and Zhang 2019) and from Constraint Programming (CP) (Hu et al. 2019; Verhaeghe et al. 2019).

Another question is whether these DTs are pure (i.e., classify correctly the training dataset). To avoid overfitting on the training set, and therefore get lower accuracy on the test set, there is a rule of thumb that makes us prefer some impurity to get more generalization power. However, it is difficult to anticipate how much impurity we should allow.

In this paper, we first address the question of how to efficiently compute minimum pure DTs and discuss later how accurate they are. We focus on CP techniques where SATisfiability (SAT) (Biere et al. 2009) based approaches have been shown to be very effective. Particularly, we will work on the Maximum Satisfiability (MaxSAT) problem, which is the optimization version of the SAT problem.

We revisit the SAT approach in Narodytska et al. (2018) which encodes into SAT the decision query of whether there is a pure DT of exactly *n* nodes. Then, by performing a sequence of such SAT queries the optimum can be located. We extend this SAT approach to the MaxSAT case, i.e., we encode into MaxSAT the query representing which is the minimum number of nodes *n* for which such a pure DT exists.

Equipped with a MaxSAT encoding we can now leverage the power of current (and *future*) MaxSAT solvers. Notice that the MaxSAT formalism allows us to state explicitly that we are dealing with an optimization problem, while the original incremental SAT solving approach in Narodytska et al. (2018) is agnostic in this sense and the objective has to be managed by an *ad hoc* piece of code. Our experimental results show that our approach in practice is able to obtain minimal pure DTs on a wide variety of benchmarks faster than the original SAT incremental counterpart in Narodytska et al. (2018) even using the same underlying SAT solver. We also show that our approach provides comparable performance to other approaches (Janota and Morgado 2020) that exploit explicitly structural properties of the DT.

Secondly, and the main contribution of the paper, we show that by exploring the space of optimal solutions (minimal pure DTs) we can be indeed competitive with respect to DT classifiers generated with sklearn in terms of producing smaller trees but yet accurate enough.

In order to achieve the main goal of this paper, we solve yet another optimization problem that extracts multiple optimal solutions, maximizing their *diversity*, from the MaxSAT encoding that allows us to compute minimum pure DTs for the training set. Then, thanks to a validation process, we select among these multiple diverse solutions one that is expected to report better accuracy on the test set.

This is an interesting result since all the optimal solutions we consider are minimum pure DTs for the training set, yet they do not seem to suffer substantially from the *overfitting* phenomena. Notice that what makes them special is that they all are of minimum size, a feature that can not be exploited by current DT ML approaches.

Finding MPDTs is an NP-hard problem, and therefore a time-consuming process. Our goal is to provide a solution for applications where high interpretability and accuracy of DTs prevail over the training speed, such as the AI systems considered as “high-risk” in the proposed regulation (European Commission 2021). In particular, this solution is suitable for systems where a certain stability for the models (i.e. not re-training the model frequently) is desired. This would be the case of models used in administration selection processes, financial risk ratings, or patient screening. Also, having interpretable models in contraposition to black box models (such as neural networks) allows to extract knowledge from those models and analyze the model to detect potential biases.

## Preliminaries

### Definition 1

An example *e* is a set of pairs $\langle f_r,v \rangle$ plus the class label *c*, where $f_r$ is a feature and *v* is the value assigned to the feature. A Decision Tree (DT) is a set of rules (namely paths) that are constructed by traversing a tree-like structure composed of questions (decision nodes) and answers until a terminal node with a label (leaf) is found. The depth of a node is the length of the path (i.e. count of nodes traversed) from the root to this node. The depth of the DT is the length of the largest path from the root to any leaf. A Complete Decision Tree (CDT) is a tree where all the leaves are located at the same depth. A Pure Decision Tree (PDT) for a set of examples $\varepsilon$, $PDT(\varepsilon )$, is a DT that classifies correctly all the examples (Hautaniemi et al. 2005). By definition, its accuracy on this set is $100\%$. A Minimum Pure Decision Tree (MPDT) for a set of examples $\varepsilon$, $MPDT(\varepsilon )$, is a DT that is proven to be the smallest PDT for $\varepsilon$ in terms of size (i.e. the number of nodes in the DT). We denote the minimum size as $|MPDT(\varepsilon ) |$.

### Example 1

Table 1 shows the set of examples $\varepsilon$ for the meteo dataset. Column *#* shows the index of each example *e* and column *classes* their class label *c*.

In Fig. 1 we can see a Minimum Pure Decision Tree (MPDT) for the set of examples in Table 1, where the decision nodes are drawn as ellipses and the leaves as rectangles. It has 5 paths and a depth of 3. In particular, the leaves of the MPDT define the following partition of the examples, from top to bottom and left to right, $\langle \{1,4,8,9\},\{11,12,14\},\{5,7\},\{2,3,6\},\{10,13\} \rangle$. As we can see all the examples classified in a leave belong to the same class (Pure) and there is no other Pure tree with less nodes (Minimum).

**Table 1:** The meteo dataset

#	Outlook	Temperature	Humidity	Windy	Classes
1	overcast	hot	high	false	+
2	rain	mild	high	false	+
3	rain	cool	normal	false	+
4	overcast	cool	normal	true	+
5	sunny	cool	normal	false	+
6	rain	mild	normal	false	+
7	sunny	mild	normal	true	+
8	overcast	mild	high	true	+
9	overcast	hot	normal	false	+
10	rain	mild	high	true	-
11	sunny	hot	high	false	-
12	sunny	hot	high	true	-
13	rain	cool	normal	true	-
14	sunny	mild	high	false	-

**Fig. 1:**
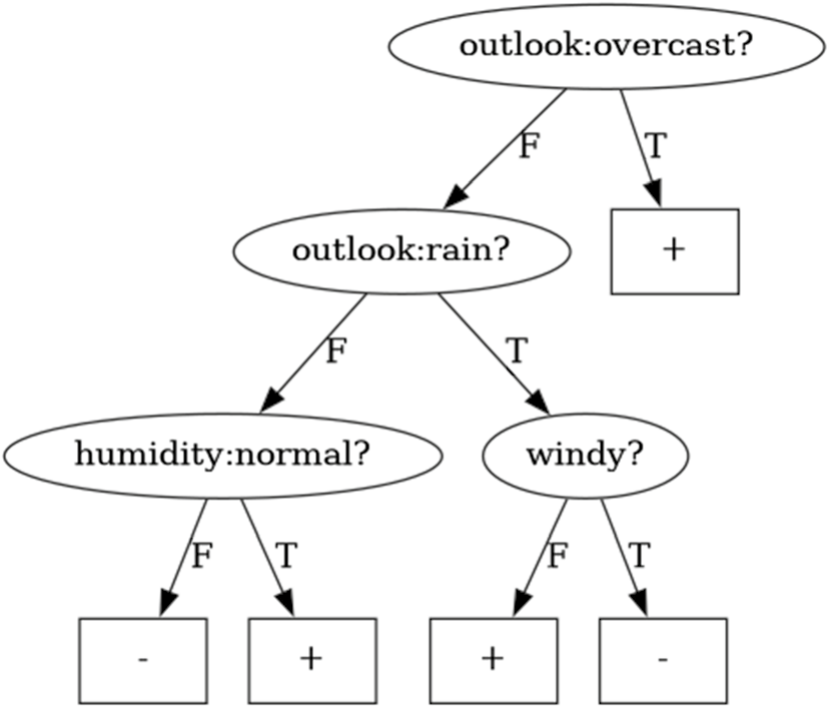
MPDT for the meteo dataset

### Definition 2

A literal is a propositional variable *x* or a negated propositional variable $\lnot x$. A clause is a disjunction of literals. A Conjunctive Normal Form (CNF) is a conjunction of clauses. A weighted clause is a pair (*c*, *w*), where *c* is a clause and *w*, its weight, is a natural number or infinity. A clause is hard if its weight is infinity (or no weight is given); otherwise, it is soft. A Weighted Partial MaxSAT instance is a multiset of weighted clauses.

### Definition 3

A truth assignment for an instance $\phi$ is a mapping that assigns to each propositional variable in $\phi$ either 0 (False) or 1 (True). A truth assignment is *partial* if the mapping is not defined for all the propositional variables in $\phi$. A truth assignment *I* satisfies a literal *x* $(\lnot x)$ if *I* maps *x* to 1 (0); otherwise, it is falsified. A truth assignment *I* satisfies a clause if *I* satisfies at least one of its literals; otherwise, it is violated or falsified. The cost of a clause (*c*, *w*) under *I* is 0 if *I* satisfies the clause; otherwise, it is *w*. Given a partial truth assignment *I*, a literal or a clause is undefined if it is neither satisfied nor falsified. A clause *c* is a unit clause under *I* if *c* is not satisfied by *I* and contains exactly one undefined literal. The cost of a formula $\phi$ under a truth assignment *I*, $cost(I, \phi )$, is the aggregated cost of all its clauses under *I*.

### Definition 4

The Weighted Partial MaxSAT problem for an instance $\phi$ is to find an assignment in which the sum of weights of the falsified soft clauses, cost($\phi$), is minimal and, the hard clauses are satisfied. The Partial MaxSAT problem is the Weighted Partial MaxSAT problem where all weights of soft clauses are equal. The SAT problem is the Partial MaxSAT problem when there are no soft clauses. An instance of Weighted Partial MaxSAT, or any of its variants, is unsatisfiable if its optimal cost is $\infty$. A SAT instance $\phi$ is satisfiable if there is a truth assignment *I*, called model, such that $cost(I, \phi ) = 0$.

### Definition 5

A *pseudo-Boolean* constraint (PB) is a Boolean function of the form $\sum _{i=1}^{n} q_il_i \diamond k$, where *k* and the $q_i$ are integer constants, $l_i$ are literals, and $\diamond \in \{<, \le , =, \ge , >\}$ is a comparison operator. An *At Least K* (AtLeastK) constraint is a PB of the form $\sum _{i=1}^{n} l_i \ge k$. An *At Most K* (AtMostK) constraint is a PB of the form $\sum _{i=1}^{n} l_i \le k$.

## Related work

In a pioneering work (Bessiere et al. 2009), the first SAT approach to compute MPDTs was presented. They encode into SAT the decision query of whether there exists a PDT of size *n*. They use as upper bound the solution from a CP model. Then, they perform a sequence of queries to a SAT solver (in the SAT4J package (Le Berre and Parrain 2010) where *n* is iteratively bounded by adding an *AtLeastK* constraint on the number of *useless* nodes that must be in the solution.

In Narodytska et al. (2018) a more efficient SAT encoding was provided to encode the decision query of whether there exists a PDT of size *n*. The authors also solve the problem through a sequence of SAT queries. In this case, the upper bound is computed with the algorithm ITI (Utgoff 1989). Then, at every iteration, they reencode the problem with the next possible value of *n*. We refer to this approach as *MinDT*.

In Avellaneda (2020) two methods are proposed. The *Infer-depth* approach iteratively decreases the depth of the tree, till it locates the minimum depth to build a complete PDT. The *Infer-size* approach computes the MPDT with the depth found with *Infer-depth*. While both approaches compute *small* PDTs, none of them guarantees to find MPDTs.

In Janota and Morgado (2020), one of the approaches, *dtfinder*, split the search space into the possible *topologies* (tree layouts) of the DT till a certain depth. At least one of these *topologies* is guaranteed to belong to an MPDT.

In Hu et al. (2020), *DT(max)*, the approach answers to which is the best tree in terms of accuracy in the training set that can be obtained given an upper bound on the depth. Note that if the upper bound is smaller than the minimal depth of MPDT, their approach reports a tree with less than 100% of accuracy on the training set (i.e. impure tree). Additionally, an AdaBoost (Freund and Schapire 1997) approach based on MaxSAT is presented. We will not explore AdaBoost strategies in this paper, since they produce ensembles, which are composed of several DTs. Having more than one DT weakens the interpretability of the classifier.

Table 2 shows a summary, presenting the objective function, whether the approach returns a PDT and if it is minimum (MPDT). The only algorithms that are complete in the sense of certifying the minimum size of the PDTs are (Bessiere et al. 2009), MinDT (Narodytska et al. 2018) and *dtfinder* (Janota and Morgado 2020).

We will focus on comparing our approach *MPDT* (see Sect.  4) with *MinDT* in terms of computing efficiently MPDTs since other approaches build on *MinDT*. In terms of computing rather *small* trees with good accuracy, we will compare with *DT(max)* (Hu et al. 2020) which in contrast to the other approaches focuses also on maximizing the accuracy.

**Table 2:** State-of-the-art: SAT-based approaches for computing DTs

Algorithm	Objective	PDT	MPDT
*Bessiere et al.*	min. size	yes	yes
*MinDT*	min. size	yes	yes
*Infer-depth*	min. depth	yes	no
*Infer-size*	min. depth, size	yes	no
*dtfinder*	min. size	yes	yes
*DT(max)*	max. acc	no	no
MPDT	min. size	yes	yes

## Computing MPDTs with MaxSAT

We extend the SAT encoding in Narodytska et al. (2018), MinDT, to a Partial MaxSAT (and its weighted variant) encoding that queries for an MPDT given an upper bound in size of *n*. Our approach is inspired by the MaxSAT encoding in Ansótegui et al. (2013) for constructing minimum covering arrays. Recent work in Hu et al. (2020) follows a similar approach with the $m_i$ variable, which is used to relax all the constraints of the encoding. We selectively use the relevant variable only in the constraints that require it. For completeness and the aim of reproducibility, we describe in full detail the original SAT encoding and the extensions to the MaxSAT case, highlighting the changes from the original encoding.

We assume that: (1) the features are binary; (2) the root node is labeled as node 1 (the rest of them are labeled into breadth-first order); and (3) positive (negative) answers to decision nodes are assigned to the right (left) child. In Tables 3 and 4 we show the symbols, functions, and the propositional variables of the encoding.

**Table 3:** Symbols and functions used in the MaxSAT encoding

Sym/fun	Definition
$\varepsilon$	The set of observations used to train the tree
$\varepsilon ^{+(-)}$	The set of positive (negative) observations
*n*	The upper bound on the number of nodes
*N*	Range of nodes, starting at 1
$N^e$	Range of even-indexed nodes
$N^o$	Range of odd-indexed nodes
*k*	Number of features for each observation, starting at 0
*F*	Range of features for each observation, starting at 0
*lr*(*i*)	Set of potential left child nodes for node *i*
*rr*(*i*)	Set of potential right child nodes for node *i*
$\sigma (r,q)$	The value of the feature $f_r$ at observation $e_q$
$\eta (i)$	Related variable $\eta _{i'}$ for the node *i*

**Table 4:** Variables used in the MaxSAT encoding

Variable	Index’s Range	Represents
$v_i$	$i \in N$	True iff. node *i* is a leaf
$l_{i,j}$	$i \in N, j \in lr(i)$	True iff. node *j* is the left child of node *i*
$r_{i,j}$	$i \in N, j \in rr(i)$	True iff. node *j* is the right child of node *i*
$p_{j,i}$	$i \in N, j \in N$	True iff. node *i* is the parent of node *j*
$\eta _i$	$i \in N^o$	True iff. nodes *i* and $i-1$ are used
$a_{r,j}$	$r \in F, j \in N$	True iff. feature $f_r$ is assigned to node *j*
$u_{r,j}$	$r \in F, j \in N$	True iff. feature $f_r$ has been discriminated in the path between the root and node *j*
$d^v_{r,j}$	$v \in \{0, 1\}, r \in F, j \in N$	True iff. feature $f_r$ is discriminated for value *v* by node *j* or one of its ancestors
$c_j$	$j \in N$	True iff. class assigned to node *j* is 1
$\lambda _{t,i}$	$i \in N, t \in [0, {\lceil }\frac{i}{2}{\rceil }]^{a}$	True iff. there are (at least) *t* leaves until (and included) node *i*
$\tau _{t,i}$	$i \in N, t \in [0, i]$	True iff. there are (at least) *t* decision nodes until (and included) node *i*

$^{a}$ In Narodytska et al. (2018) it is stated that $t \le {\lfloor }\frac{i}{2}{\rfloor }$. This does not hold for the last node of a complete tree

We add a new variable $\eta _i$ which represents if a node *i* is used. The encoding works on an upper-bounded number of nodes. If the upper bound is not optimal, some nodes will not be used. Since nodes are numbered in breadth-first order, two consecutive nodes with the same parent will be both in the solution or not. Therefore, we define function $\eta (i)$ as:

$$\begin{aligned} {} \eta (i) = {\left\{ \begin{array}{ll} \eta _{i+1} &{} \textrm{if}\; i \in N^e; \\ \eta _{i} &{} \textrm{if}\; i \in N^o \end{array}\right. } \end{aligned}$$

The soft clauses in the Partial MaxSAT encoding that describe the objective function of the MPDT problem are:

$$\begin{aligned} {} \bigwedge _{i \in N^{o}}(\lnot \eta _i, 1)\qquad \qquad \qquad \qquad ({ SoftN}) \end{aligned}$$

Now, we describe the hard constraints. To encode PB constraints we use OptiLog (Ansótegui et al. 2021).

**Node usage hard clauses:**

• If an odd node is not used, then both himself and the previous one are not leaves.

1 $$\begin{aligned} { \bigwedge _{i \in N^o\ |\ i\ > 1} \lnot \eta _i \rightarrow \lnot v_i \wedge \lnot v_{i-1} } \end{aligned}$$

• If a node is used, the previous ones are used:

2 $$\begin{aligned} { \bigwedge _{i \in N^o\ |\ i\ > 1} \eta _i \rightarrow \eta _{i-2} } \end{aligned}$$

• To enforce a lower bound *lb* (3 by default) we add the following clause.

3 $$\begin{aligned} {\eta (lb)} \end{aligned}$$

**Tree layout hard clauses:**

- The root node is not a leaf.
  4 $$\begin{aligned} \lnot v_1 \end{aligned}$$
- (i) Leaves do not have a left child, and (ii) if node *i* is used in the DT, and has no left child candidates, then it is a leaf.
  5 $$\begin{aligned} \bigwedge _{i \in N} {\left\{ \begin{array}{ll} \bigwedge _{j \in lr(i)} (v_i \rightarrow \lnot l_{i,j})) &{} {\textrm{if}\ \vert lr(i)\vert \ne 0}\\ {\eta (i) \rightarrow v_i} &{} {\textrm{if}\ \vert lr(i)\vert = 0} \end{array}\right. } \end{aligned}$$
- Node *j* is the left child of node *i* iff node $j+1$ is the right child of node *i*.
  6 $$\begin{aligned} \bigwedge _{i \in N} \bigwedge _{j \in lr(i)} (l_{i,j} \leftrightarrow r_{i,j+1}) \end{aligned}$$
- If a used node *i* is not a leaf, then it has exactly one left child.
  7 $$\begin{aligned} \bigwedge _{i \in N} \left( (\lnot v_i {\wedge \eta (i)}{}) \rightarrow \left( \sum _{j \in lr(i)} l_{i,j} = 1 \right) \right) \end{aligned}$$
• The symmetry equations on $p_{j,i},l_{i,j}$ and $r_{i,j}$.
  8 $$\begin{aligned} \begin{aligned} \bigwedge _{i \in N} \bigwedge _{j \in lr(i)} (p_{j,i} \leftrightarrow l_{i,j}) \\ \bigwedge _{i \in N} \bigwedge _{j \in rr(i)} (p_{j,i} \leftrightarrow r_{i,j}) \end{aligned} \end{aligned}$$
  Given a pair of *i*, *j* nodes, we have that $j \in lr(i) \cup rr(i)$, and $lr(i) \cap rr(i) = \emptyset$. This, combined with Constraint 8, sets an equivalence between the variable $p_{j,i}$ and one of $l_{i,j}$ or $r_{i,j}$ depending on the value *j* being even or odd. Thus, in practice, we can perform a direct substitution of the variable $p_{j,i}$ with the corresponding one of those two variables in the encoding, and remove the $p_{j,i}$ variable and the Constraint 8 altogether. In this description, for clarity purposes, we kept the $p_{j,i}$ variable in the encoding.
- If node *j* is used in the DT (and it is not the root) it will have exactly one parent.
  9 $$\begin{aligned} \bigwedge _{j \in N\ |\ j > 1} \left( {\eta (j) \rightarrow }{} \sum _{i={\lfloor }\frac{j}{2}{\rfloor }}^{j-1} p_{j,i} = 1 \right) \end{aligned}$$

**Feature assignment hard clauses:**

• Feature $f_r$ with value 0 (1) is already discriminated by node *j* iff the feature $f_r$ is attached to the parent of *j* and node *j* is the right (left) child or the feature $f_r$ with value 0 (1) is already discriminated by the parent of *j*.

10 $$\begin{aligned}&\bigwedge _{j \in N, r \in F} \left( d_{r,j}^{0} \leftrightarrow \bigvee \limits _{i={\lfloor }\frac{j}{2}{\rfloor }}^{j-1} \left( (a_{r,i} \wedge r_{i,j}) \vee (p_{j,i} \wedge d_{r,i}^{0}) \right) \right) \end{aligned}$$

11 $$\begin{aligned}&\bigwedge _{j \in N, r \in F} \left( d_{r,j}^{1} \leftrightarrow \bigvee \limits _{i={\lfloor }\frac{j}{2}{\rfloor }}^{j-1} \left( (a_{r,i} \wedge l_{i,j}) \vee (p_{j,i} \wedge d_{r,i}^{1}) \right) \right) \end{aligned}$$

• No feature is discriminated at the root node.

12 $$\begin{aligned} \bigwedge _{r \in F} \left( \lnot d_{r,1}^{0} \wedge \lnot d_{r,1}^{1} \right) \end{aligned}$$

• If some feature $f_r$ is already discriminated by node *i*, then the feature can not be assigned to any of its children.

13 $$\begin{aligned} \bigwedge _{j \in N, r \in F} \bigwedge \limits _{i={\lfloor }\frac{j}{2}{\rfloor }}^{j-1} ((u_{r,i} \wedge p_{j,i}) \rightarrow \lnot a_{r,j}) \end{aligned}$$

• Feature $f_r$ is discriminated at node *j* iff $f_r$ has been assigned to node *j* or it is already discriminated at the parent of *j*.

14 $$\begin{aligned} \bigwedge _{j \in N, r \in F} \left( u_{r,j} \leftrightarrow \left( a_{r,j} \vee \bigvee \limits _{i={\lfloor }\frac{j}{2}{\rfloor }}^{j-1} (u_{r,i} \wedge p_{j,i})\right) \right) \end{aligned}$$

• If node *j* is not a leaf and it is in the DT, then it has exactly one feature assigned to it.

15 $$\begin{aligned} \bigwedge _{j \in N} \left( (\lnot v_j {\wedge \eta (j)}{}) \rightarrow \sum _{r \in F} a_{r,j} = 1 \right) \end{aligned}$$

• If node *j* is a leaf, then it has no feature assigned to it.

16 $$\begin{aligned} \bigwedge _{j \in N} \left( v_j \rightarrow \sum _{r \in F} a_{r,i} = 0 \right) \end{aligned}$$

• For every positive (negative) example, if node *j* is a leaf and it is assigned to class 0 (1), then at least one feature is discriminated with the value in the example at node *j*.

17 $$\begin{aligned} \bigwedge _{j \in N, e_q \in \varepsilon ^{+}} \left( (v_j \wedge \lnot c_j) \rightarrow \bigvee \limits _{r \in F} d_{r,j}^{\sigma (r,q)} \right) \end{aligned}$$

18 $$\begin{aligned} \bigwedge _{j \in N, e_q \in \varepsilon ^{-}} \left( (v_j \wedge c_j) \rightarrow \bigvee \limits _{r \in F} d_{r,j}^{\sigma (r,q)} \right) \end{aligned}$$

**Additional Pruning hard clauses:** Depending on which nodes are decided to be leaves or not, the possible list of children for a given node may change. These constraints enforce this simplification. We refer to the original description (Narodytska et al. 2018). We incorporate as in previous constraints the $\eta (i)$ variable to take into account in counters $\tau _{t,i}$ and $\lambda _{t,i}$ only the nodes that are used in the solution.

19 $$\begin{aligned}&\bigwedge _{i \in N} \lambda _{0,i} \wedge \tau _{0,i} \end{aligned}$$

20 $$\begin{aligned}&\bigwedge _{i \in N, t \in [1, {{\lceil }\frac{i}{2}{\rceil }}{}]} ( \lambda _{t,i} \leftrightarrow ( \lambda _{t,i-1} \vee \lambda _{t-1,i-1} \wedge v_i {\wedge \eta (i)}{} ) ) \end{aligned}$$

21 $$\begin{aligned}&\bigwedge _{i \in N, t \in [1, i]} ( \tau _{t,i} \leftrightarrow ( \tau _{t,i-1} \vee \tau _{t-1,i-1} \wedge \lnot v_i {\wedge \eta (i)}{} ) ) \end{aligned}$$

22 $$\begin{aligned}&\bigwedge _{i \in N, t \in [1, {{\lceil }\frac{i}{2}{\rceil }}{}]} \lambda _{t,i} \rightarrow \lnot l_{i, 2(i-t+1)} \wedge \lnot r_{i, 2(i-t+1) + 1} \end{aligned}$$

23 $$\begin{aligned}&\bigwedge _{i \in N, t \in [{\lceil }\frac{i}{2}{\rceil }, i]} \tau _{t,i} \rightarrow \lnot l_{i, 2(t - 1)} \wedge \lnot r_{i, 2t - 1} \end{aligned}$$

### Multilabel encoding

We extend *MinDT* to support multilabels where $c_{k,j}$ variables now represent that the leaf *j* belongs to class $k \in Cls$.

24 $$\begin{aligned} {} \bigwedge _{k \in Cls} \bigwedge _{j \in N, e_q \in \varepsilon ^{k}} \left( (v_j \wedge c_{k,j}) \rightarrow \bigvee \limits _{r \in K} d_{r,j}^{\sigma (r,q)} \right) \end{aligned}$$

• A decision node has no class assigned:

25 $$\begin{aligned} {} \bigwedge _{j \in N, k \in Cls} (\lnot v_j \rightarrow \lnot c_{k,j}) \end{aligned}$$

• A leaf has only one label:

26 $$\begin{aligned} {} \bigwedge _{j \in N}\left( v_j \rightarrow \sum _{k \in Cls} = 1\right) \end{aligned}$$

### Partial MaxSAT encoding

We assume a dataset $\varepsilon$, an upper (*ub*) and lower (*lb*) bounds on the size of the tree. Then:

#### Proposition 1

Let $PMSat_{PDT}^{\varepsilon ,n=ub,lb}$ be $SoftN \wedge (eq.1) \wedge \ldots \wedge (eq.26)$. The optimal cost of the Partial MaxSAT instance $PMSat_{PDT}^{\varepsilon ,n=ub,lb}$ is ${\lceil }\frac{|MPDT(\varepsilon )|}{2}{\rceil }$.

## The *Linear* MaxSAT algorithm

We present an slightly variation of the Linear SAT-based MaxSAT algorithm (Eén and Sörensson 2006; Berre and Parrain 2010) that we will use to solve the Partial MaxSAT encoding presented in Sect. 4.

Essentially, Algorithm 1 solves a (Weighted) Partial MaxSAT optimization problem through a sequence of SAT queries. It receives as input parameters a (Weighted) Partial MaxSAT formula $\phi$ (a set of *soft* $\phi _s$ and *hard* $\phi _h$ clauses), an optional timeout *t*, and an optional upper bound. First, we create an incremental SAT solver *s* (line 1). Then, we add all the hard clauses $\phi _h$ and a copy of the soft clauses each extended with a new *blocking* variable $b_i$ (line 2). Then, the upper bound *ub* of $\phi$ is computed as the sum of the weights $w_i$ in $\phi _s$ plus one (line 3). An *incremental* PB encoder (an encoder which its *k* can be updated) is initialized in line 4. This PB encoder initially restricts the sum of the $b_i$ variables and their associated weights $w_i$ to be $\le ub$. In line 5, we use the PB encoder to translate into CNF the initial PB constraint. While this PB constraint in combination with the hard constraints is satisfiable, we extract the model, update the *ub* to the cost of this model and refine the PB constraint (lines 7–12). Finally, we return the upper bound *ub*, the model *m*, and a reference to the incremental SAT solver *s*.
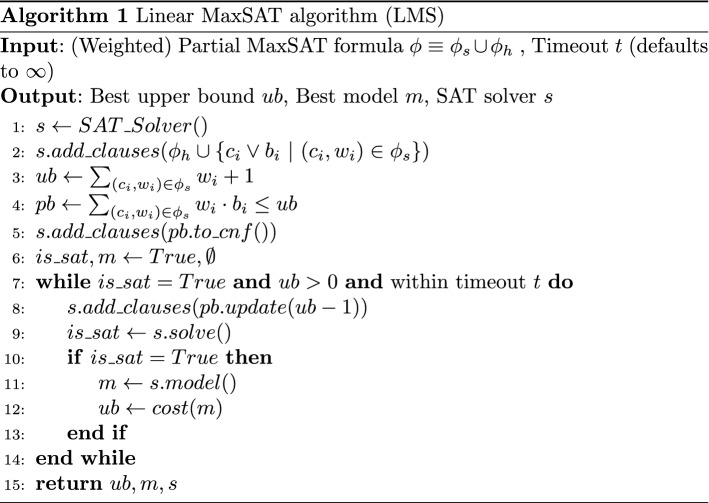


## Extracting multiple diverse solutions

We can obtain multiple solutions of a given optimal cost *c* by generating the SAT instance representing the query of whether there is a solution of cost *c*. Then, we execute a SAT solver in incremental mode on the SAT instance, and whenever we get a solution, we add its negation to the SAT solver and ask the solver to solve the augmented instance.

In our approach, we take advantage of the fact that the SAT instance representing the optimal solutions is already created during the execution of the Linear MaxSAT algorithm which may be also augmented by useful learned clauses added in previous calls to the SAT solver. In particular, it is the last satisfiable SAT instance seen by Linear MaxSAT.

To be able to access to the clauses of this last satisfiable instance we modify the Linear MaxSAT algorithms described in Algorithm 1 as follows. First, we move the statement of line 8 ($s.add\_clauses(pb.update(ub-1))$) inside the conditional block ($is\_sat = True$) of line 10 and add as an assumption to the solver in line 9 the auxiliary variables that reify the clauses in $pb.update(ub-1)$.^1^ At this point, the formula containing all the possible optimal solutions corresponds to *s*.*clauses*().

However, these solutions may be too *similar*. In our case, we expect that diverse solutions (diverse MPDTs) may help to get a more robust approach in terms of accuracy.

To enforce this diversity we will solve another MaxSAT problem. Algorithm MDSOL (Alg. 2) shows the pseudocode to extract multiple diverse solutions. As input we have the SAT formula that compiles the solutions, the number of solutions we want to extract, and the *target* vars on which we will enforce our diversity criterion.
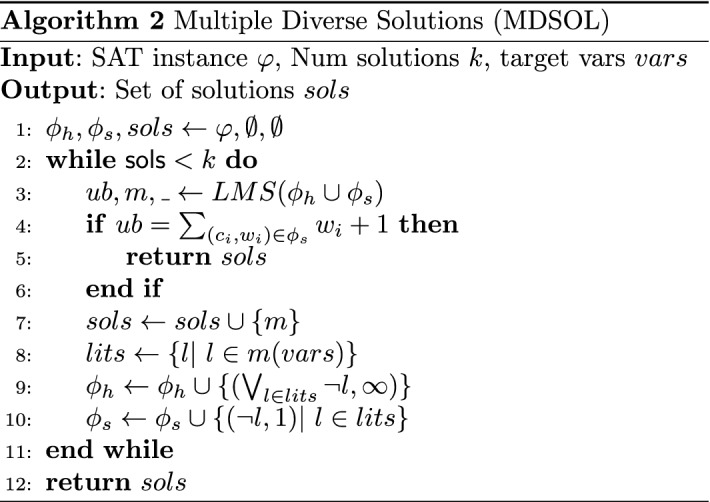


The set of hard clauses of the MaxSAT formula we iteratively solve contains the clauses of the input SAT instance and the clauses discarding the solutions extracted so far (line 8) restricted to the target variables.

The soft clauses contain, as unit clauses of cost 1, the negation of the literals (restricted to the target variables) appearing in the solutions retrieved so far (line 10). This way we prefer solutions where the polarity of the target variables appears less in the previous retrieved solutions. Notice that eventually, this approach can return all the optimal solutions.

## Computing minimum pure decision trees

Finally, algorithm MPDT (Alg.3) describes how we compute MPDTs for a given dataset. We split randomly the input dataset into training and selection datasets, where *p* is the percentage for examples to add to the training set (line 1).

In line 3 we perform a local search on the training dataset to find an upper bound on the tree size. In particular, we use ITI (Utgoff 1989) (see Sect. 9) to find a pure decision tree (that is not guaranteed to be minimum).

Then, we create the MaxSAT encoding for computing MPDTs, described in Sect. 4 (line 4), on the training set $\varepsilon _{tr}$ and the lower and upper bounds.

In line 5 we call the MaxSAT solver. We assume that as in the Linear MaxSAT pseudocode the MaxSAT solver returns a container for the SAT clauses compiling all the optimal solutions.

In line 6 we call MDSOL (Alg. 2) that extracts *k* multiple *diverse* MPDTs. We add as target vars the $a_{r,j}$ vars which represent that feature *r* is assigned to node *j*. Preliminary experiments showed that using this variable is central and that adding other variables such as $u_{r,j}$ or $v_i$ does not lead to better results.

We consider also a variation, MPDT_lay, inspired on Janota and Morgado (2020). In short, we identify the $k'$ tree layouts to a certain depth that can lead to an MPDT. Then, we compute for each of these $k'$ layouts $k/k'$ solutions through MDSOL algorithm.

From lines 8 to 10, we add to $best_{dts}$ the MPDTs such that their accuracy, evaluated on the selection set $\varepsilon _{sel}$ is greater than or equal the best accuracy in $best_{dts}$ minus a deviation percentage $\delta$ (line 10). Finally, we return the set $best_{dts}$. In this paper, we just take randomly one of the MPDTs from $best_{dts}$ but other solutions combining more than one MPDT can be applied as other well-known approaches in machine learning (e.g. random forests). 
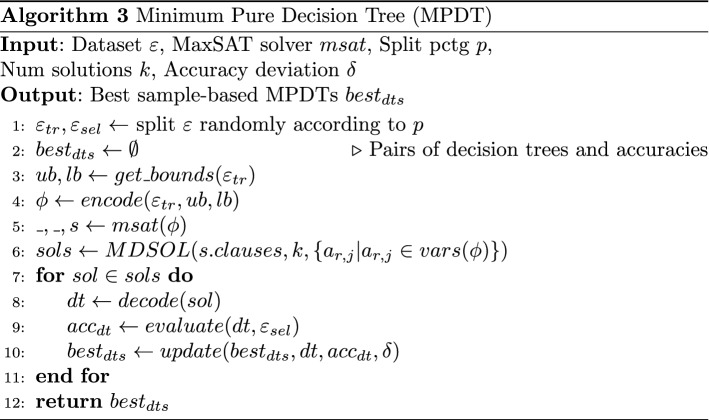


## Preprocesing datasets

We use the |KBinsDiscretizer| transformer from sklearn to convert the continuous variables into discrete values using 8 bins (intervals of values) with the uniform strategy (same amount of values for each range). Then, we convert all the non-binary features to binary using our custom binary encoding that applies a mapping from the original domain to a numeric domain. If the feature is ordinal, the order is kept. Then, those numeric values are represented in binary, and a new feature is created for each bit required to encode the entire domain for the original feature. Thus, for a feature with a domain *D* we replace it with $log_2(|D |)$ new features. As described in Narodytska et al. (2018), the size of the encoding grows in ${\mathcal {O}}(kn^2+mnk)$, with *k* being the number of features. Note that the binary encoding should have a natural advantage in the encoding size compared to the typical one-hot encoding (OHE)^2^ for non-binary datasets (see Table 5).

Finally, for each dataset benchmark, we split (following the stratification strategy) the dataset into a 64% training set, 16% selection set, and 20% test set.

## Experimental results

As benchmarks we use datasets (binary and non-binary) from the CP4IM repository (De Raedt et al. 2008), the UCI repository (Dua and Graff 2017), and the PennML repository (Romano et al. 2021; Olson et al. 2017). Table 5 shows their statistics. Our execution environment consists of a computer cluster with nodes equipped with two AMD 7403 processors (24 cores at 2.8 GHz) and 21 GB of RAM per core.

**Table 5:** Statistics from the datasets used in the experimental results

	#Columns		#Labels	#Rows			
	Binary	OHE		Total	Train	Sel	Test
append	22	56	2	105	67	17	21
audio	146	146	2	216	137	35	44
back	50	89	2	180	115	29	36
corral	7	7	2	160	102	26	32
haber	11	28	2	266	169	43	54
hayes-r	9	16	3	120	76	20	24
hepa	58	321	2	155	99	25	31
h-votes	17	17	2	435	278	70	87
iris	13	32	3	150	96	24	30
irish	16	53	2	500	320	80	100
lympho	30	51	4	148	94	24	30
monks-1	11	16	2	556	355	89	112
monks-3	11	16	2	548	350	88	110
mushr	112	112	2	8124	5199	1300	1625
mux6	7	7	2	128	81	21	26
new-thy	16	39	3	212	135	34	43
spect	23	23	2	251	160	40	51
wine	47	263	3	178	113	29	36
zoo	26	122	7	101	64	16	21

In order: the number of features for both the binary encoding and the OHE, the number of labels (2 for binary classification instances, more for multilabel), the total number of rows in the dataset, and the number of rows used as train, selection, and test

### On MPDT runtime

The first question we address is whether the MPDT MaxSAT encoding described in Sect. 4 in combination with a MaxSAT solver allows us to compute faster MPDTs than the *MinDT* approach described in Narodytska et al. (2018).

For this, we adapted MinDT and the Linear algorithm (Algorithm 1) used to solve the MaxSAT encoding to report the intermediate results found during the search process. Both of them are set to use the SAT solver CaDiCaL (Biere 2019) as the SAT oracle. Then, we compare those approaches between them and against the best incomplete solvers on the unweighted track of the 2021 MaxSAT evaluation (Bacchus et al. 2021). In particular, we use the incomplete solvers *SATLike-c* (Lei et al. 2021), *SATLike-ck* (Lei et al. 2021), and *TT-Open-WBO-Inc-21* (Nadel 2021).

We set a time limit of 1h and a memory limit of 20GB. We focused on large datasets (rows/features) and took 20 samples at each percentage $S_{pctgs}=\{40\%, 60\%, 80\%\}$, for a total of 580 instances for each size. This is a relevant experiment since if one wants to scale to large datasets as in Schidler and Szeider (2021) using a kind of Large Neighborhood Search approach, it needs to efficiently solve samples of the original dataset with a SAT-based approach. Given these limits, we are interested in which approach is able to report the best upper bound (i.e. the smallest pure tree) on the given instances. For completeness, we also run the same experiment on the $80\%$ samples with a timeout of 10h.

The *ub* for each instance is computed using the same approach as Narodytska et al. (2018), i.e. the upper bound is the size of the computed $PDT(\varepsilon _{spl})$ using ITI algorithm (Utgoff 1989). The *lb* is trivially set to 3.

Table 6 reports, for each sample size (column “Sample size”), the number of instances (column “# Best UB”) where each approach (column “Solver”) is able to report the best upper bound on the instance. Column “Encoding” indicates which encoding is used.

**Table 6:** Number of instances where the best upper bound was reported by each incomplete solver

Sample size	Timelimit	Solver	Encoding	# Best UB
40%	1h	satlike-ck	MaxSAT	528
40%	1h	MPDT	MaxSAT	505
40%	1h	satlike-c	MaxSAT	504
40%	1h	TT-Open-WBO-Inc-21	MaxSAT	489
40%	1h	MinDT	SAT	469
60%	1h	satlike-ck	MaxSAT	491
60%	1h	satlike-c	MaxSAT	426
60%	1h	MPDT	MaxSAT	421
60%	1h	TT-Open-WBO-Inc-21	MaxSAT	417
60%	1h	MinDT	SAT	404
80%	1h	satlike-ck	MaxSAT	422
80%	1h	satlike-c	MaxSAT	383
80%	1h	MPDT	MaxSAT	404
80%	1h	TT-Open-WBO-Inc-21	MaxSAT	382
80%	1h	MinDT	SAT	374
80%	10h	satlike-ck	MaxSAT	510
80%	10h	satlike-c	MaxSAT	405
80%	10h	MPDT	MaxSAT	425
80%	10h	TT-Open-WBO-Inc-21	MaxSAT	406
80%	10h	MinDT	SAT	400

As we can see, the *satlike-ck* solver is the best solver, while *MinDT* performs the worst in this experiment. This indicates that using the MaxSAT encoding allows for a faster reduction of the bounds in the MPDT problem.

Notice that MaxSAT algorithms share learnt clauses during the entire optimization process, do not require generating the SAT query at each step, and can skip suboptimal bounds. This is not the case for SAT incremental approaches as *MinDT*, which can explain the differences that we observe in the results.

This experiment confirms it is useful to solve optimization problems as MaxSAT instances rather than with an agnostic SAT incremental approach provided memory requirements hold.

Notice anyway that the main goal of our paper is addressed in the next experiments, where most of the runtime is consumed on extracting multiple solutions rather than on computing the first MPDT.

### On MPDT accuracy

The second question we want to address is how accurate can be the MPDT approach described in Sect. 6. We compare with the DecisionTreeClassifier model from sklearn (Scikit-learn developers 2020). For these experiments, we set a time limit of 1 day and 100GB of memory.

We recall that datasets are split into 64% training set, 16% selection set, and 20% test set. We try exactly 50 different random splits of the selection and test sets, select the best DT (from those returned by the particular approach) on the selection split and report the average accuracy on the test set.

For MPDT we evaluate *mptd-1* that only generates one solution, and the variations that extract multiple solutions (with no diversity enforcement) (*mpdt-nd*), multiple diverse solutions (*mpdt*). Following Janota and Morgado (2020), we also adapted our algorithm to consider the explicit *layouts* for the decision trees, having *mpdt-lay-nd* for the version that does not enforce diversity, and *mpdt-lay* for the diverse solutions algorithm. This experiment considers the complete version of the MPDT algorithm.

For sklearn, we evaluate three variations on the training set with as many seeds as DTs returned by the MPDT version with more solutions. In pure sklearn (*psk*) we run sklearn till it achieves 100% accuracy. In limited sklearn (*lsk*) we restrict the DT size to the one reported by MPDT. Finally, in All sklearn (*ask*) we explore each possible DT size from 3 to the maximum size reached by the *psk* version. Notice that in *ask* we evaluate many more DTs than for the previous approaches. Notice that *lsk* and *ask* may not compute Pure DTs in contrast to MPDT and *psk*.

**Table 7:** Test accuracy score

Dataset	psk		lsk	ask		opt	mpdt-nd		mpdt-lay-nd		mpdt-1	mpdt		mpdt-lay	
	t.acc	size	t.acc	t.acc	size		#sols	t.acc	#sols	t.acc	t.acc	#sols	t.acc	#sols	t.acc
append	70.48	27	66.29	65.43	13	21	984	70.00	1167	70.00	70.00	592	**71.05**	984	70.00
audio	92.45	17	92.45	93.35	7	17	30000	93.95	30000	92.77	**94.27**	48	93.50	4685	93.36
back	69.67	33	74.28	**74.44**	3	25	5714	70.50	880	70.44	69.83	8	72.00	39	71.61
corral	100	27	87.38	100	27	**13**	20	99.25	20	99.25	**100**	20	99.25	20	99.25
haber	80.26	73	77.33	79.51	25	47	624	79.00	1173	79.00	**80.74**	216	79.00	624	79.00
hayes-r	79.33	41	77.00	77.08	31	35	1195	**83.25**	1722	**83.25**	78.08	349	82.92	1195	**83.25**
hepa	60.84	27	60.84	64.83	17	23	1695	**67.16**	86	65.87	62.32	7	64.77	18	62.77
h-votes	93.31	41	94.00	93.98	15	33	11522	**94.34**	21007	**94.34**	94.25	370	93.56	11085	**94.34**
iris	94.53	27	**94.53**	91.46	19	**25**	30000	94.53	30000	94.53	92.80	436	94.53	28280	94.53
irish	100	15	97.94	99.28	13	**9**	6	**100**	6	**100**	**100**	6	**100**	6	**100**
lympho	**72.93**	37	70.87	71.07	37	27	4214	71.73	5213	71.73	70.07	455	71.80	4214	71.73
monks-1	**100**	29	**100**	**100**	29	29	2368	**100**	2496	**100**	**100**	423	**100**	2368	**100**
monks-3	98.25	45	98.22	**99.45**	15	41	29952	99.11	30000	99.11	97.44	296	99.11	9050	99.11
mush	100	23	99.97	99.97	23	**15**	444	**100**	477	**100**	**100**	444	**100**	444	**100**
mux6	90.85	37	58.69	88.53	37	**15**	2	**100**	2	**100**	**100**	2	**100**	2	**100**
new-thy	92.60	33	**92.60**	92.14	31	**29**	30000	91.35	30000	91.35	89.58	423	91.35	18584	91.35
spect	77.37	57	76.98	71.06	3	35	288	**87.53**	369	**87.53**	**87.53**	288	**87.53**	288	**87.53**
wine	90.28	31	**95.33**	92.78	27	23	32	92.44	32	92.44	92.67	32	92.44	32	92.44
zoo	91.24	17	91.24	91.24	17	17	9364	91.24	9885	91.24	89.76	740	**94.76**	9364	91.24
mean	87.07		84.15	86.61				88.70		88.57	87.86		**88.82**		88.50
median	91.24		91.24	91.46				92.44		92.44	92.67		**93.50**		92.44

Size of the best solution is shown for non-optimal methods

Table 7 shows the average accuracy for each benchmark. For *psk* and *ask* we show the size of the DT (which may not be optimal). Additionally, for each dataset, we highlight in bold the approach with the best accuracy (ties are broken by DT size).

As we can see, extracting multiple diverse solutions (*mpdt*) helps to improve the average accuracy of the MPDT approach. Moreover, with respect to sklearn, *mpdt* obtains an average improvement on accuracy of 1.75%, 4.7% and 2.2% with respect to *psk*, *lsk* and *ask*.

Therefore, on the same size *mpdt* shows a much better accuracy-interpretability tradeoff than sklearn. Moreover, it is remarkable that *mpdt* using much fewer DTs than *ask*, and using Pure DTs, it is yet able to get a better average result. It seems that minimizing the size of PDTs helps to mitigate the overfitting phenomena. In this sense, it is interesting to explore the interactions with other properties such as the robustness of DTs (Moshkovitz et al. 2021).

Additionally, it is clear that *mpdt* misses some good solutions, in particular, the first one used by *mpdt-1*. Therefore, we also studied, whether any approach was able to *see* a DT with better accuracy on the test set but was not chosen with the selection set. In particular, *mpdt* obtains an average of 91.31%, *psk* 89.31%, *lsk* 86.65% and *ask* 90.51%.

We also compare with *DT(max)* (Hu et al. 2020)^3^ which uses the incomplete MaxSAT solver Loandra (Berg et al. 2020). Although Loandra is (according to the results of the MaxSAT evaluation (Bacchus et al. 2021) superior to the Linear MaxSAT solver we use, our approach *mpdt* still reports better results than *DT(max)* using, as in the original paper, depth limits of 2, 3 and 4 (see Sect. 3). In particular, we find improvements of 7.86%, 4.37%, and 1.69% respect to *DT(max)* limited on depths 2, 3, and 4 respectively.

Finally, although in this paper we focus on the trade-off between interpretability versus accuracy we may wonder how is the performance in terms of accuracy of sklearn on the original datasets prior to discretizing the continuous features in the preprocessing step described in Sect. 8. We conducted this experiment as in Table 8 and we observed that although *ask* and *psk*^4^ improve their mean (88.10% and 88.4%, respectively) and median (92.56% and 93.31%) performance they do not beat the *mpdt* approach which additionally guarantees the minimum size of the DT. Certainly, as future work, a promising research line is to study how to discretize better the continuous parameters to improve both the size of the DT and its accuracy through the mpdt approaches.

**Table 8:** Test accuracy score compared to SKlearn using the binary encoding and no encoding at all vs. complete approaches with the binary encoding

Dataset	PSK		ASK		MPDT-nd	MPDT-lay-nd	MPDT-1	MPDT	MPDT-lay	Mean
	No enc.	Bin	No enc.	Bin						
append	**79.71**	70.48	78.76	65.43	70.00	70.00	70.00	71.05	70.00	71.71
audio	92.45	92.45	93.27	93.35	93.95	92.77	**94.27**	93.50	93.36	93.26
back	66.89	69.67	**78.00**	74.44	70.50	70.44	69.83	72.00	71.61	71.52
corral	100	100	100	100	99.25	99.25	**100**	99.25	99.25	99.67
haber	78.25	80.26	77.30	79.51	79.00	79.00	**80.74**	79.00	79.00	78.95
hayes-r	**83.41**	79.33	79.08	77.08	83.25	83.25	78.08	82.92	83.25	81.14
hepa	67.61	60.84	**74.26**	64.83	67.16	65.87	62.32	64.77	62.77	65.68
h-votes	93.31	93.31	93.98	93.98	**94.34**	**94.34**	94.25	93.56	**94.34**	93.93
iris	94.67	94.53	**96.07**	91.46	94.53	94.53	92.80	94.53	94.53	94.03
irish	**100**	100	**100**	99.28	100	100	100	100	100	99.92
lympho	**72.40**	72.93	69.86	71.07	71.73	71.73	70.07	71.80	71.73	71.33
monks-1	**100**	100	**100**	100	100	100	100	100	100	100
monks-3	98.83	98.25	98.82	99.45	**99.11**	**99.11**	97.44	**99.11**	**99.11**	98.76
mush	100	100	99.97	99.97	**100**	**100**	**100**	**100**	**100**	99.99
mux6	90.85	90.85	88.54	88.53	**100**	**100**	**100**	**100**	**100**	95.42
new-thy	**93.67**	92.60	84.65	92.14	91.35	91.35	89.58	91.35	91.35	91.45
spect	77.37	77.37	71.06	71.06	**87.53**	**87.53**	**87.53**	**87.53**	**87.53**	81.61
wine	92.39	90.28	92.56	92.78	92.44	92.44	**92.67**	92.44	92.44	92.46
zoo	**95.90**	91.24	**95.90**	91.24	91.24	91.24	89.76	94.76	91.24	92.50
MEAN	88.30	87.86	88.00	86.61	88.70	88.57	87.86	**88.82**	88.50	
MEDIAN	92.45	91.85	92.56	91.46	92.44	92.44	92.67	**93.50**	92.44	

## Conclusions and future work

We have presented an efficient approach for computing MPDTs providing new insights on the accuracy-interpretability tradeoff. We have shown that by computing MPDTs for the training set, we can also get in practice accurate DTs on the test set compared to DT heuristic approaches in ML. This is a curious result since technically we overfit on the training set. As future work, we will explore further the relation of the size of the tree and its pureness on the training set in terms of prediction accuracy on the test set. We will also explore how to produce better multiple optimal solutions from our MaxSAT approach and combine them effectively to construct other predictors.

## Data Availability

Data and software are available upon request.

## Footnotes

1 If $pb.update(ub-1)$ is a unit clause, we just add its literal as an assumption.

2 The OHE encoding leads to worse results.

3 DT(max) is comparable to DL8.5 (Aglin et al. 2020) which improves (Verhaeghe et al. 2019).

4 *lsk* can not be applied since the optimal size for datasets with continuous parameters is not known.
